# A stochastic model for affect dynamics: methodological insights from heart rate variability in an illustrative case of Anorexia Nervosa

**DOI:** 10.3389/fpsyt.2025.1502217

**Published:** 2025-02-25

**Authors:** Francesca Borghesi, Gloria Simoncini, Riccardo Cremascoli, Laura Bianchi, Leonardo Mendolicchio, Simone Cappelli, Federico Brusa, Stefania Cattaldo, Elisa Prina, Alice Chirico, Alessandro Mauro, Pietro Cipresso

**Affiliations:** ^1^ Department of Psychology, University of Turin, Turin, Italy; ^2^ Istituto Auxologico Italiano, Istituto di Ricovero e Cura a Carattere Scientifico (IRCCS), Unit of Neurology and Neurorehabilitation, San Giuseppe Hospital Piancavallo, Verbania, Italy; ^3^ Istituto Auxologico Italiano, IRCCS, Experimental Laboratory for Metabolic Neurosciences Research, San Giuseppe Hospital Piancavallo, Verbania, Italy; ^4^ Istituto Auxologico Italiano, IRCCS, Laboratorio di Psicologia, Ospedale S. Giuseppe, Verbania, Italy; ^5^ Istituto Auxologico Italiano, IRCCS, Laboratory of Clinical Neurobiology, San Giuseppe Hospital Piancavallo, Verbania, Italy; ^6^ Department of Neurosciences "Rita Levi Montalcini", University of Turin, Turin, Italy; ^7^ Laboratory of Clinical Neurobiology, IRCCS Istituto Auxologico Italiano, San Giuseppe Hospital, Verbania, Italy; ^8^ Department of Psychology, Research Center in Communication Psychology, Universitá Cattolica del Sacro Cuore, Milan, Italy; ^9^ Department of Neuroscience Rita Levi Montalcini, University of Turin, Turin, Italy

**Keywords:** affect dynamics, heart rate variability, Markov chain, psychometrics, stochastic model, neuroscience, Anorexia Nervosa

## Abstract

**Background:**

Affect dynamics, or variations in emotional experiences over time, are linked to psychological health and well-being, with moderate emotional variations indicating good psychophysical health. Given the impact of emotional state on cardiac variability, our objective was to develop a quantitative method to measure affect dynamics for better understanding emotion temporal management in Anorexia Nervosa (AN).

**Methods:**

The study proposed an experimental and methodological approach to evaluate physiological affect dynamics in clinical settings. It tested affective transitions and temporal changes using emotional images from the International Affective Picture System (IAPS), examining physiological characteristics of a patient with AN. The methodology involved calculating a heart rate variability index, e.g., RMSSD, and using it in a Discrete Time and Discrete Space Markov chain to define, quantify, and predict emotional fluctuations over time.

**Results:**

The patient with Anorexia Nervosa showed a high likelihood of transitioning from positive to negative emotional states, particularly at lower arousal levels. The steady state matrix indicated a tendency to remain in highly activated pleasant states, reflecting difficulties in maintaining emotional balance.

**Conclusions:**

Employing Markov chains provided a quantitative and insightful approach for examining affect dynamics in a patient with AN. This methodology accurately measures emotional transitions and provides a clear and interpretable framework for clinicians and patients. By leveraging Markovian indexes, mental health professionals may gain a comprehensive understanding of emotional fluctuations’ patterns. Moreover, graphical representations of emotional transitions may enhance the clinician-patient dialogue, facilitating a clearer emotional and physiological profile for the implementation of personalized treatment procedures.

## 1 Introduction

Increasing empirical evidence supports the notion that affect dynamics - as the temporal fluctuations of our emotional experiences – which is intricately linked with well-being and psychological health (1–3). This burgeoning field of research indicates that not all emotional experiences are created equal, and the way our emotions change and flow over time can provide critical insights into our overall mental health (4–7). In general, medium variability in affective dynamism is associated with good psychophysical health; the opposite effect is observed for extreme states of too high variability or extreme rigidity (e.g., inability to switch from one affective state to another) (8–11). The extreme in affective dynamism is opposite on a descriptive level but equal from a psycho-physiological point of view and have been deeply investigated in the realm of psychiatric or neurological disorders (8, 12). Specifically, affect dynamics have been studied to gain a better understanding of how individuals with conditions such as bipolar disorder, depression, anxiety and eating disorders, experience and feel emotion in time (13–16). In eating disorders, most of all in AN, contemporary theories acknowledge the importance of disordered emotional functioning in the disorder’s development and maintenance [e.g., (17, 18)]. AN is characterized by symptoms of extreme mental and emotional rigidity, making it a compelling case for studying issues in emotion regulation and affect dynamics. Individuals with AN often exhibit a pronounced inability to adaptively manage and transition between emotional states, reflecting significant challenges in emotional flexibility. This rigidity is manifested in both cognitive and emotional domains, leading to a limited range of emotional experiences and heightened difficulty in coping with negative affect (10, 19, 20). Most recent studies have focused on affective variability- pattern of frequent and large mood shifts over time- or on its opposite affective inertia- emotions that are resistant to change or shifts over time (18, 21, 22). The study of Vansteelandt and colleagues (17) investigated affect variability using Experience Sampling Method protocol in patient with AN restricted and Bulimia Nervosa (BN). Results of this exploratory study suggest that the diagnostic groups have the same mean levels of affect, but the AN restricted group showed a less variability in the quality of their affect. Recently, also Williams-Kerver et al. (23) demonstrated, using Experience sampling Method, that participants in the AN and BN groups experienced significantly greater Negative Affect (NA) intensity and better emotion differentiation- the ability to discriminate distinct emotional states- than participants in the Binge Eating Disorders (BED) group. Alternatively, the BN group demonstrated significantly greater NA variability than the AN group and greater NA inertia than the BED group.

All studies described relied most on the Russell’s circumplex model (24), and the predominant experimental design methodology was Ecological Momentary Assessment (EMA) (25). This method allowed collecting real-time data in naturalistic settings, providing a valuable window into individuals’ emotional lives (26–28). EMA involves prompting patients to report on their feelings, behaviors, and environmental context at random times throughout the day, thereby offering a rich, detailed account of affect dynamics (29).

Although EMA is an ecological and longitudinally accurate instrument, it necessitates high participant compliance, that is, the person must be able (and willing) to respond to most of the responses over time. Furthermore, EMA can only delimit and describe the long-term time series of affective states reported by the participant’s responses, assessing mostly the mood; it cannot provide information about short time affective transitions that the person can make during the day or week. Finally, to the best of our knowledge, no one study assesses the physiological characteristic of affect dynamics in eating disorders using EMA, may be due to the compliance and the difficulty of the continuous physiological signals (30–32). Incorporating Heart Rate Variability (HRV), particularly RMSSD, as a physiological signal for affect dynamics is supported by extensive research linking it to autonomic nervous system function and emotional regulation (33). Studies by Thayer et al. (34) and Schmalbach et al. (35) demonstrate that HRV is a robust indicator of emotional regulation and stress resilience, underscoring its relevance for psychological and neurological assessments. Particularly, research by Appelhans and Luecken (36) highlights RMSSD’s specificity in reflecting parasympathetic activity, making it a common measure for estimating the variations in heart rate that are mediated by the vagus nerve and therefore it represents an ideal measure for studying affective dynamics. Recent research underscores the validity of HRV, particularly RMSSD, as a physiological index in psychiatric conditions, linking it to emotional regulation and neurophysiological changes across disorders such as depression and anxiety. HRV reflects the body’s capacity for emotional and physiological regulation, with lower HRV associated with higher symptom severity in psychiatric disorders (12, 35). These findings support the use of HRV in monitoring emotional regulation, offering a rationale for its selection as a physiological index in our study on affect dynamics. Adding RMSSD as a key physiological measure in our study is further justified by its mathematical significance in quantifying short-term variations in heart rate. RMSSD stands for the Root Mean Square of Successive Differences between normal heartbeats. It’s calculated using the formula:


$$\text{RMSSD}=\;\sqrt{\frac{1}{\text{N}-1}\sum_{\text{i}=1}^{\text{N}-1}{\left(\text{R}{\text{R}}_{\text{i}+1}-\text{R}{\text{R}}_{\text{i}}\right)}^{2}}$$

where (*RR_i_*) represents the duration of one heartbeat to the next (in milliseconds), and (N) is the total number of heartbeats analyzed. This formula captures the variability in heart rate over short periods, making it an excellent index for assessing autonomic nervous system function, particularly the parasympathetic branch. RMSSD’s focus on short-term variability provides a sensitive measure of vagal tone, a key aspect of emotional and physiological regulation.

For this, reason we developed and implemented a new experimental and methodological technique to study AN’s affect dynamics with physiological signals (e.g. RMSSD) in the clinical setting. The goal is to create a simple and effective assessment of affect dynamics, investigating all possible affective transitions and to measure it with Discrete-Time and Discrete Space Markov processes. While previous techniques have focused on group-level data analysis using panel data methodologies, thus, losing sight of the individuality and uniqueness of individual emotional experiences, here, our methodology allows for a personalized, patient-centered perspective. The experimental design involves the emotive images’ visualization of the International Affective Picture System (IAPS), (37) to stimulate all possible affective transitions, based on Russell’s Circumplex model (4). HRV is measured during all the experimental session, and the RMSSD is used into this novel methodological approach of analysis based on Discrete Time and Discrete Space Markovian stochastic processes. Markov chains are mathematical systems that describe a sequence of possible events, where the probability of each event depends only on the state attained in the previous event (38). In the context of affect dynamics, Markov chains can be employed to analyze and predict emotional state transitions over time (39), utilizing physiological measures like RMSSD to understand the probabilistic nature of emotional fluctuations. This approach can effectively capture the dynamic and stochastic nature of emotional fluctuations over time, offering insights into how emotional states evolve (4, 8, 40, 41).

This method stands out for its potential to provide individualized and detailed insights into the emotional patterns of individuals affected by Anorexia Nervosa, offering new perspectives on the relationship between emotions and physiology in these disorders. With this approach, we aspire to develop more effective experimental and methodological tools for monitoring and intervention, enhancing the understanding and treatment of these complex conditions. Through the simple and quantitatively idiographic use of the grapho describing probability transitions, a comprehensive profile can be drawn up that is easy for the patient and the clinician to interpret.

## 2 Materials and methods

### 2.1 Participant

A female patient with Anorexia Nervosa with restricts behavior was selected as study’s participant. She is hospitalized at the IRCCS Istituto Auxologico Piancavallo for two weeks.

The patient affected by Anorexia Nervosa (AN) was a 35-year-old woman. She was admitted at the Unit of Eating Disorders of Istituo Auxologico Italiano in Piancavallo (Italy) in December 2023 because of severe malnutrition (Body mass index 9.7 kg/m2). She has suffered from AN since she was 19 years old. The neuropsychological evaluation showed a good mood stability with the ongoing therapy: Aripiprazole 5 mg/day, Delorazepam 1 mg/ml 5gtt+10gtt+25gtt/day, Sertraline 50 mg/day, Trazodone 60mg/ml 15gtt/day. During the 4 weeks of hospitalization the patient underwent a rehabilitation program based on refeeding in a medical setting. The refeeding program is implemented by a multidisciplinary team of doctors, dietitians, educators, and psychologists, with daily individual meetings.

The patient also went through a standardized rehabilitation program directed by a physiotherapist through exercises to improve her walking ability and endurance, and balance control an hour a day, from Monday through Saturday.

She was not given the planned pharmacological therapy close to the administration of the emotional stimuli and detection of physiological parameters.

The study was conducted in accordance with the Declaration of Helsinki, having been approved by the Ethics Committee of IRCCS Istituto Auxologico Italiano (Prot. 2022_10_25_05).

### 2.2 Inclusion criteria

The participant gave written informed consent before participating in the study. In particular, to be included in the study, participant criteria were:

- Normative performance on three neuropsychological tests, respectively phonemic (Corrected Score = 33,16) and semantic fluency (Corrected score = 50,97, (42), Trail Making Test (Part A corrected score = 39; Part B corrected score = 71; Part B-A corrected score = 32), (43) and Tower of London Test (Time Score = 28; Accuracy score = 33), (44).
- Absence of additional concomitant pathological conditions, thus, achieving results within the expected range on neuropsychological tests investigating general cognitive functioning (MMSE: score = 25 with a cut-off = 21, (45), short-term and long-term mnestic abilities (Digit Span Forward: corrected score = 5,50, (46); Rey Auditory Verbal Learning Test: Immediate recall corrected score = 32; Deferred recall corrected score = 5,40, (47) attentional capacities (TMT Test: part A corrected score = 67; (43) and executive functions (Clock Drawing Test corrected score = 10,50, (48); Frontal Assessment Battery corrected score = 14,49, (49); Coloured Progressive Matrixes corrected score = 23,50, (50).

The psychometric tools were administered to confirm normative cognitive functioning and exclude conditions that could confound the study’s focus on affect dynamics. These assessments ensured the observed patterns were specific to Anorexia Nervosa.

### 2.3 Procedure

**Figure 1:**
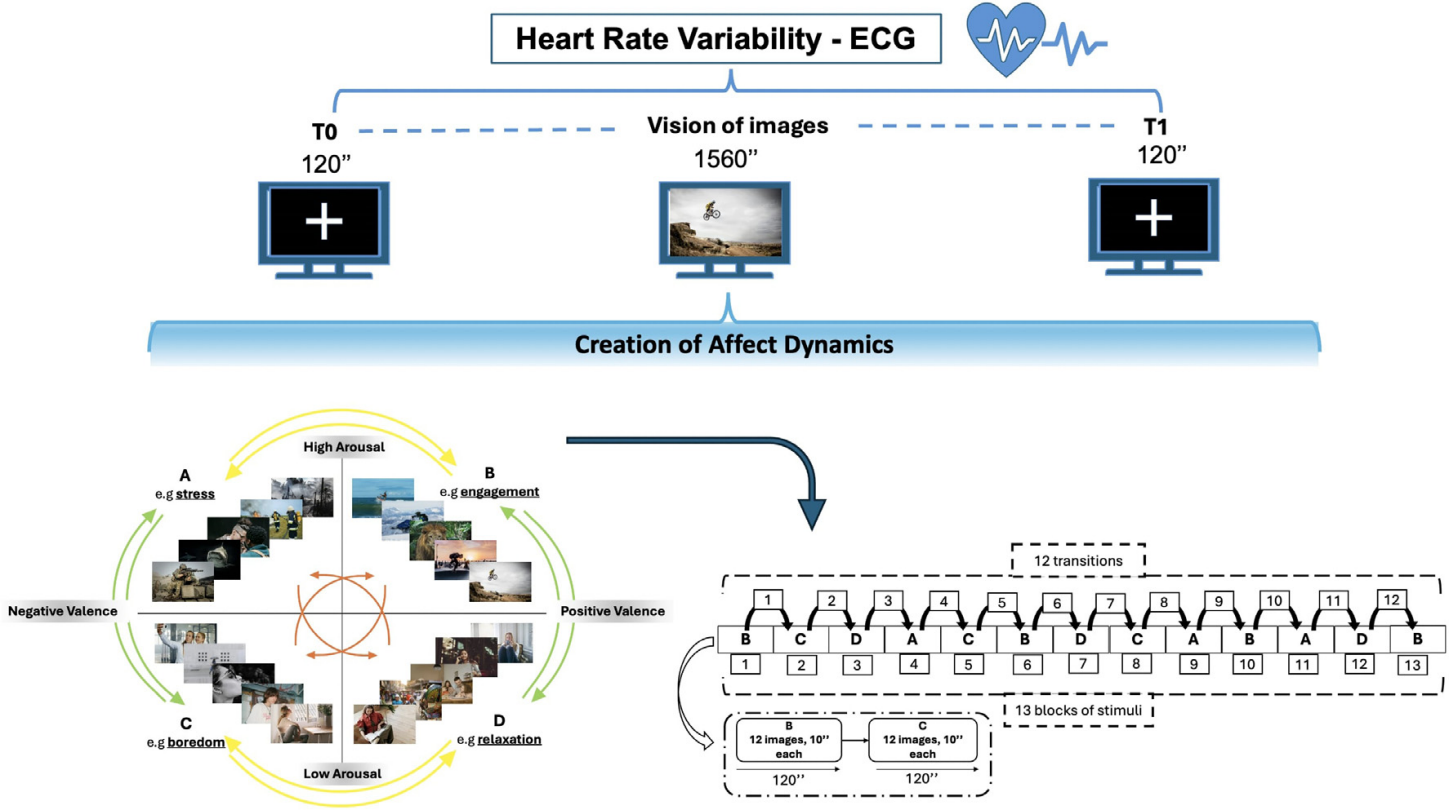
Experimental Design: Cardiac activity is recorded using ECG while observing IAPS images. There are 12 possible transitions between affective states, leading to affect dynamics. The transitions consist of horizontal movements (AB-CD-BA-DC) denoted by yellow arrows, vertical movements (AC-BD-CA-DB) shown by green arrows and diagonal movements (AD-BC-DA-CB) denoted by orange arrows.

The study followed a within-subject design to examine affect dynamics using affective induction through IAPS (37). In accordance with Russel’s Circumplex model, a total of twelve transitions were established between distinct blocks of arousal-valence (51). These transitions encompass horizontal, vertical, and oblique movements (see **Figure 1**). The visual stimuli were presented via Desktop monitor on a 42-inch screen while sitting comfortably in a chair with armrests and headrests to minimize head and arm movements, which inject noise into the data captured by the sensors. We positioned the chair about one meter from the monitor. The participant was exposed to 156 images from the IAPS dataset, arranged into 13 blocks of 2 minutes, mimic 12 transitions between arousal and valence states, as outlined by Russel’s Circumplex model (26 total minutes equivalent to 1560 seconds). Each block contains 12 images, displayed for 10 seconds, with images selected based on high (>6) or low (<4) arousal/valence Likert scores from the Self-Assessment Manikin (SAM) (52). The participant had a unique random images sequence, to avoid temporal or ceiling effect in emotional response. This design ensured a comprehensive exploration of emotional transitions, with the sequence of image presentation randomized to avoid order effects.

During the entire experimental session, heart rate variability was measured, with electrocardiogram (ECG). A baseline phase was run before and after each experimental session, observing a fixed crossing for 2 minutes (120 seconds), taking account intra-variability measure of the participant.

### 2.4 Recording of psychophysiological signals

The data on the autonomic nervous systems were collected by measuring physiological responses, i.e., Electrocardiogram. Embletta MPR acquired these responses. The responses were then processed with custom software developed using MATLAB 9.13.0 (R2023a) (The Mathworks, Inc.; Natick, MA, USA). Every channel was acquired synchronously at 1000 Hz. Visual inspection is used to the artefact correction option, instead artefacts due to ectopic beats, missed beat detections, etc. are corrected adapting an appropriate window correction level (threshold for detecting artefact beats) which removes the artefacts but does not distort normal RR intervals.

### 2.5 Psychophysiological signal processing

For ECG analysis, cardiovascular analysis is focused on the RR interval, representing the time between successive heartbeats, was used to derive heart rate (HR) and assess autonomic function. This conversion provided an HR mean (in beats per minute) and an equivalent RR mean (calculated as 60,000/HR), offering insights into heart rate variability and cardiovascular health.

RMSSD is a key time-domain measure used in heart rate variability (HRV) analysis to quantify the short-term variability of heart rate, reflecting the beat-to-beat variance in heart rate. It is calculated by taking the square root of the average of the squared differences between successive normal heartbeats over a specified period. This measure is primarily used to estimate vagally mediated changes reflected in HRV, making it a valuable index for assessing autonomic nervous system function, especially parasympathetic activity. RMSSD is considered a reliable indicator of the autonomic nervous system’s resilience and adaptability to stress. While typically a 5-minute recording period is conventional for HRV analysis, recent research supports the validity of using ultra-short-term periods, such as 10 seconds, for RMSSD calculation, providing a practical approach for both clinical and research settings. This adaptability in measurement duration facilitates the efficient assessment of autonomic function in various contexts, emphasizing RMSSD’s utility in capturing the dynamic nature of autonomic nervous system activity related to emotional and physiological states (53–55).

### 2.6 Data analysis with Markovization process

To better understand the complex and ever-changing nature of emotional transitions, we used Discrete Time and Discrete Space Markov chains, a mathematical framework for modeling sequential processes with intrinsic transitions (38, 56, 57). The changes are not entirely deterministic, but instead are regulated by probability distributions. A Discrete-time and Discrete space Markov chain is characterized by a set of states and a transition matrix P. We call a discrete-states stochastic processes a sequence of random variables X_0_, X_1_, … X_n_ where each X_n_ is a discrete random variable taking values in a set *S*, called the state space. The set *S* is finite or at most countably infinite. Without losing generality, we assume that *S* is a subset of the relative integers ℤ .

The transition matrix P for a Markov chain with X_n_ states is an n×n matrix where each element P represents the probability of transitioning from state i to state j in one discrete step (58). Inside the transition matrix, the most important Markovian property is conditional probability that the system transitions to state x_n+1_ at time n+1, given that it was in state x_n_ at time n. The term P_ij_ denotes how the system evolves over time based on its current state (59).


$$\text{P}\left({\text{X}}_{\text{n}+1}={\text{x}}_{\text{n}+1}|\;{\text{X}}_{\text{n}}={\text{x}}_{\text{n}}\right)={\text{P}}_{\text{ij}}$$

Essentially, the future of the system is determined by its current state rather than the specific route it took to get that condition. Note that we define this probability as a function of just i and j, but of course, it could depend on n as well. The time homogeneity restriction mentioned in the previous footnote is just the assumption that this probability does not depend on the time n, but rather remains constant over time.

The use of Markov chains is inspired by their ability to express probabilistic transitions between discrete states - in this case stress (A), engagement (B), boredom (C), and relaxation (D), where the probability of each event depends only on the state attained in the previous event.

In the context of affect dynamics, Markov chains can be employed to analyze and predict emotional state transitions over time, utilizing physiological measures like RMSSD to understand the probabilistic nature of emotional fluctuations (40). RMSSD is one of the most suitable indexes for heart rate variability in short term: it aligns perfectly with the properties of Markovian chains, a stochastic model of transition, which considers pairs of transitions one at a time, disregarding the path preceding that transition (60, 61).

**Figure 2:**
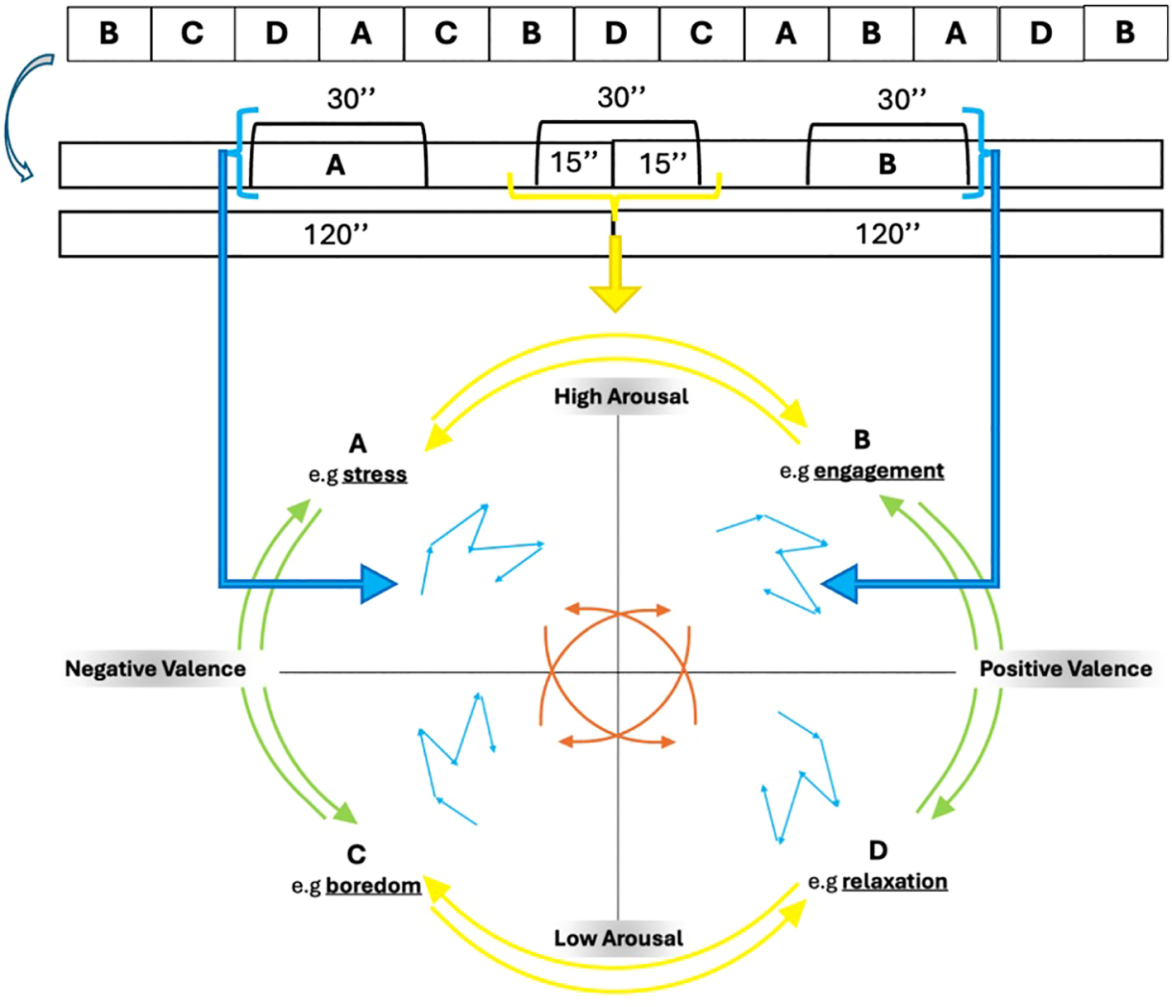
Example of transition between affective state (A, B) and the inside variability of each quadrant.

The Markov chain uses a transition matrix as its primary tool for describing the probability of moving between those affective states: It is quadratic matrix, in this case a 4*4 matrix, considering the 4 affective states. Hence, 12 elements represent probability of transition, and 4 self-transition indexes represent the inside variability of four quadrants (e.g., AA-BB-CC-DD). The 12 transition values could be divided in vertical transition (e.g., AC-CA-BD-DB), horizontal one (e.g., AB-BA-CD-DC), oblique one (AD-DA-BC-CB) (**Figure 2**).

Hence, we are able to describe all possible affective transitions of the participant, considering the outward and return of each, and also the variability inside each affective state (**Figure 2**). To express the variability between and within states, we considered the 30 seconds between two transitions (120 ± 15) and the 30 seconds between the first blocks presented by each state (60 ± 15).

The Markov chain, due to its stochastic property, necessitates that the sum of each row in the transition matrix is equal to 1:


$$\sum_{\text{j}=1}^{\text{n}}{\text{P}}_{\text{ij}}=1\text{ for all i}$$

**Figure 3:**
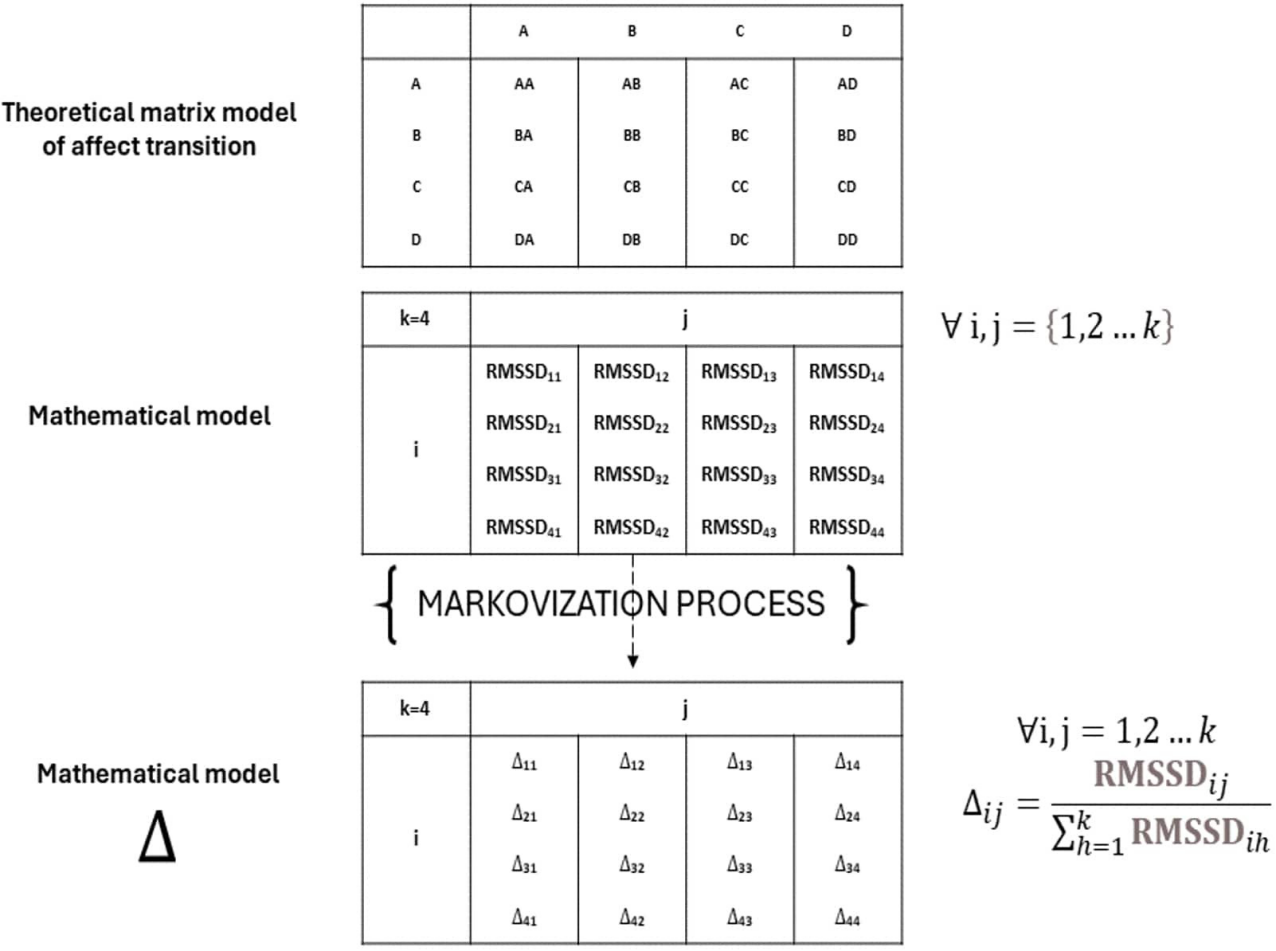
Markovization process.

For this reason, each RMSSD for 12 transitions is relativized and included into the Markov chain (Δ indexes). These transition probabilities within each row denote the likelihoods of transitioning from a given state to all feasible states within the system (**Figure 3**).

Furthermore, the Markov chain is also a predictive property, calculating the steady states vector values. The steady state distribution provides insights into the long-term behavior of the system, offering insights into the equilibrium distribution of states after a large number of transitions.

The steady states iterative calculation is not arbitrary but are significantly influenced by two factors: the initial state vector ( π _0_) and the participant-specific transition matrix (P). The initial state vector ( π _0_) posits an *a priori* probability of the participant’s presence in one of the four quadrants, serving as the starting point for the Markov chain: i.e. the participant before being subjected to stimuli is equally likely to be in one of the four affective states.

For each state I ∈ S, we denote by π_0_(i) probability P{X_0_=i} that the Markov chain starts out in state i. Formally, π_0_ is a function taking S into the interval [0,1] such that:


$$\pi_{0}\left(\text{i}\right)\;\geq \;0\text{ for all i}\in \text{S},\;\sum_{\text{i}\in \text{S}}\pi_{0}\left(\text{i}\right)\;=1$$

Equivalently, instead of thinking of π_0_ as a function from S to [0,1], we could think of π_0_ as the vector whose i-th entry is ${\text{π}}_{0}$ (i)=P{X_0_=i}. This is the probability distribution of the Markov chain at time 0.

Our initial vector considered equiprobability between initial affective states (P_A_=0.25, P_B_=0.25, P_C_=0.25, P_D_=0.25). Through iterative multiplication (10 steps) of the initial states vector ( $\pi_{0})$ and the transition matrix (P), we found the steady states. To see this numerically, we compute $\pi_{0}\;*\;P^{n}$ for increasing n to check for convergence to steady states (π).

Thus, the steady states serve as an updated version of the original matrix, modifying the initial equilibrium based on the empirical affective transitions experienced by the participant: They describe the likelihood of discovering one of the four states after attempting ten different hypothetical transitions. The iterative process between the initial states vector of probability and the transition matrix, culminating in the steady states, underscores the dynamic interplay between predisposition and experience in shaping the affective journey of individuals. This calculation of steady states is not the only method available (e.g., eigenvalue method, also used in “process data” in the **Supplementary Materials**’ section), but we believe it is the most accessible and readily applicable. All methods used have the same results. Finally, In the **Supplementary Material**, we provide a detailed explanation of all algorithms and the methodology used to calculate Discrete Time and Discrete Space Markov Chains.

A comprehensive Word file is available, guiding users on how to implement the experimental design and compute transitions for physiological data using a Discrete Time and Discrete Space Markov Chain approach.

## 3 Results

**Figure 4:**
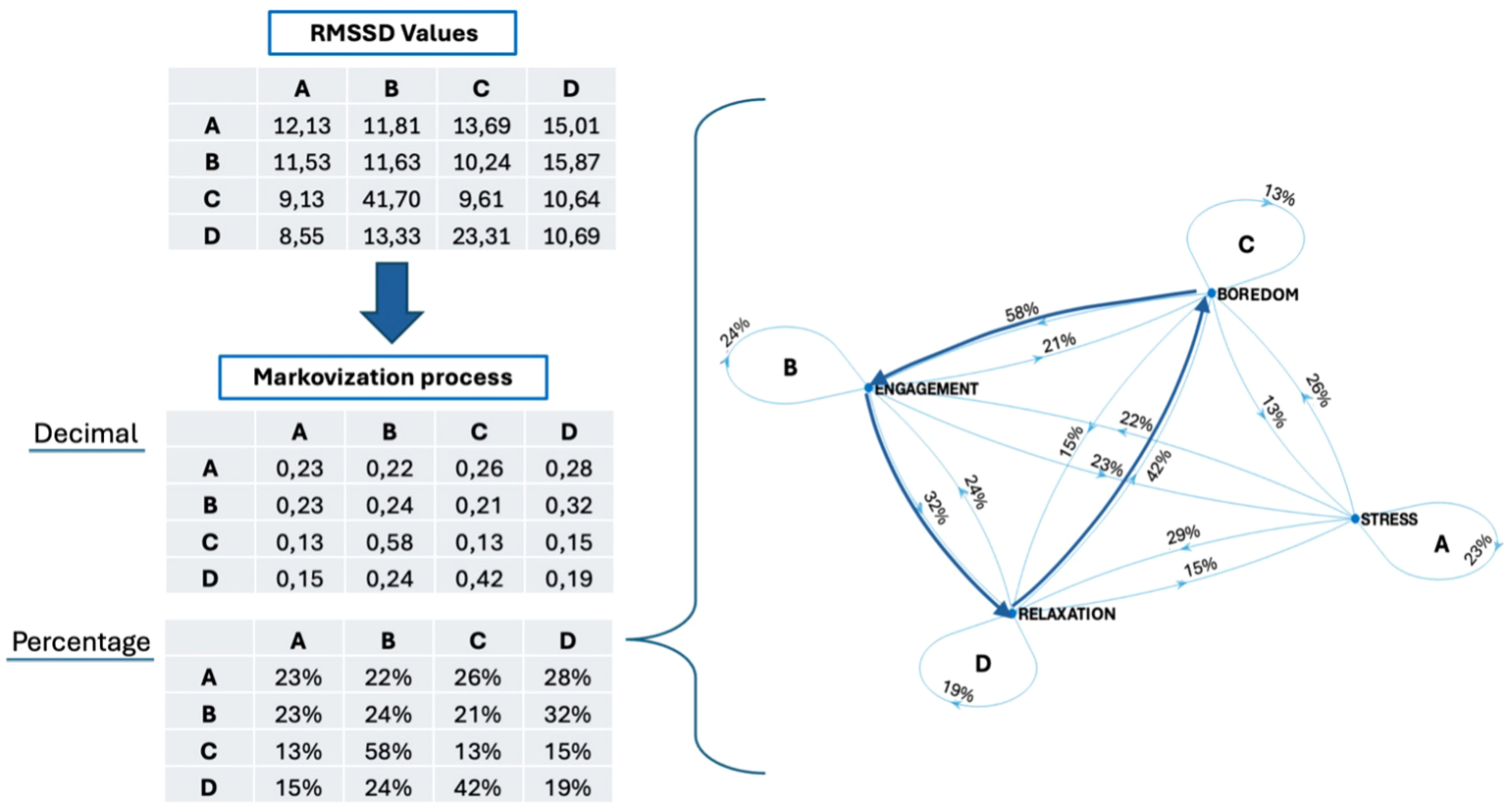
RMSSD values and Markovization processes (expressed in both decimal form and as a percentage) of the AN patient, with also its mathematical grapho representation.

The results are based on the Markovization process, which translates the variability measured by RMSSD into probabilistic terms. The RMSSD is calculated in milliseconds, for each 30’’ transition and for each singular 30’’ self-transition. This approach assesses the participant’s likelihood of variation during transitions between different affective states and the variability within the self-transition (e.g., AA-BB-CC-DD). The **Figure 4** presented the value of RMSSD of each transition, before and after the Markovization process.

After a Markovization process, we computed twelve RMSSD transitional indexes (vertical, horizontal, oblique), four static self-transition indexes, and four predictive indexes of steady state. Vertical probability indexes (AC-CA-BD-DB) represent the percentage of transitions between affective states with the same valence levels but opposing arousal levels (high-to-low and vice versa). The horizontal probability indexes (AB-BA-CD-DC) show changes between emotional states that have the same level of arousal but differ in valence (from positive to negative and vice versa). The oblique indexes (AD-DA-BC-CB) indicate the likelihood of transitions between emotional states that involve changes in both arousal and valence. The self-transition indexes (AA-BB-CC-DD) suggest that the participant’s affective state is consistent and unchanging. Steady states represent the likelihood of a person being in a specific emotional state after n step, hypothetically moving between the four quadrants ten steps.

**Figure 5:**
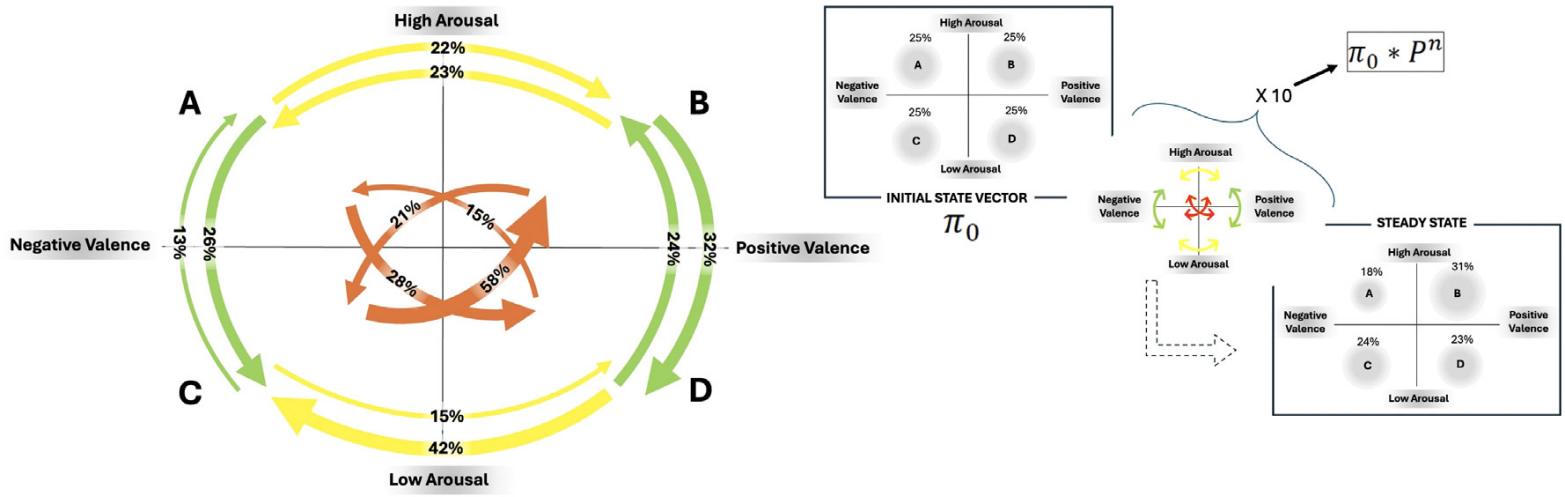
On the left side: Descriptive Markov chain containing transition values: Green arrows represent vertical transitions, yellow arrows depict horizontal transitions, and orange arrows signify oblique transitions. The percentage probability of RMSSD transitioning between states is indicated on each arrow. On the right side: Predictive Markov chain with initial state vector (π_0_) and steady state after 10 steps.

Upon analyzing **Figure 5**, discrepancies in the probability of moving from one emotional state to another were noticeable.

Self-transition indexes indicate that the individual with anorexia is more likely to maintain an activated (both positive and negative) emotional state compared to other states ( P _AA_ = 0.23; P _BB_ = 0.24; P _CC_ = 0.13; P _DD_ = 0.19).

Vertical transitions from high to low activation exhibited a higher probability (for both degrees of valence) than the opposite ones ( P _AC_ = 0.26; P _CA_ = 0.13; P _BD_ = 0.32; P _DB_ = 0.24).

When transitioning horizontally, there was a clear difference in the probability of moving from positive to negative states compared to the reverse. Particularly, during periods of low arousal, shifts from positive emotions to negative states were more probable ( P _AB_ = 0.22; P _BA_ = 0.23; P _CD_ = 0.15; P _DC_ = 0.42).

The oblique shifts showed more imbalances in transitioning between the four quadrants, with a greater likelihood of moving from highly activated negative states to low-activated positive states and from low-activated negative states to highly activated positive states ( P _AD_ = 0.28; P _DA_ = 0.15; P _BC_ = 0.21; P _CB_ = 0.58).

Observing **Figure 5**, it is also possible to state that the participant is predicted to experience a significant activation state with positive valence following repeated shifts between emotional states, as indicated by the steady state prediction vector ( P _A_ = 0.18; P _B_ = 0.31; P _C_ = 0.24; P _D_ = 0.23).

## 4 Discussion

Although the impact of affective dynamics in well-being and health has been studied (62), addressing the physiological layer of this process, also involving an AN patient, poses technical and methodological issues. Our experimental and methodological aim was to develop a patient-centered assessment of dynamism of affective states. First, we assessed the physiological correlate of affect dynamics during the viewing of emotional pictures from the IAPS database, using a laboratory experimental design. Then, using RMSSD indexes into a Discrete-Time and Discrete Space Markov Chain, we were able to compute the probability of transitioning between various emotional states or remaining in a specific state.

Utilizing the Markov chain grapho, a quantitative idiographic approach, we noticed that the patient with anorexia nervosa exhibited particular patterns of behavior. When experiencing lower levels of arousal, the patient was 41% more likely to switch from positive emotional states (such as relaxation) to negative ones (such as boredom), showing strong physiological reactivity. This finding corresponds to other research that emphasizes the difficulties that persons with AN have in regulating their emotions and often encountering heightened negative affect (NA) when exposed to stimuli (18, 63). In conditions of heightened arousal, more balance was observed. This could be attributed to the body’s efforts to maintain homeostasis during stress or engagement, resulting in more regulated emotional responses. Functional MRI studies show that during high arousal, there is increased regulation in brain regions responsible for emotional control, supporting the observation of more balanced emotional states in these conditions (64).

Vertical axis showed increased variability during the shift from high to low activation in conditions with positive valence. Some transitions showed extremely reduced probabilities, particularly when transitioning from low to high activation levels.

As oblique transitions were considered, there was a strong likelihood that the patient would shift from negative to positive emotional states. Observations differed from the outcomes in horizontal transitions, which showed a higher likelihood of moving from positive to negative affective states. To transition from a negative to a positive affective state, the patient needed to regulate their level of activation by switching between low and high arousal. This trend aligns with prior research, which demonstrates the maladaptive techniques for regulating emotions in individuals with AN, such as suppression and avoidance. The need for extreme changes in emotion regulation may suggest the presence of maladaptive methods (18).

The self-transition indexes revealed a greater probability of the patient staying in quadrant B, which represented highly engaged positive emotional states. The likelihood of staying in the other quadrants diminished, resulting in lower variability and reduced sensitivity to these conditions. This observation is consistent with research findings that suggest individuals with anorexia nervosa demonstrate increased functional connectivity in certain brain regions linked to emotional regulation, such as the dorsolateral prefrontal cortex (DLPFC), while simultaneously displaying decreased connectivity in other regions. This disparity adds to their emotional inflexibility and the inclination to persist in certain emotional states for extended durations, thereby reducing the range and responsiveness to shifting emotional circumstances (64, 65).

The steady state matrix displayed a reduced balance among the four quadrants. After ten repetitions of emotional transitions, the patient with AN was more likely to migrate to a highly activated pleasant emotional state, followed by the possibility of experiencing low activating negative emotional states. Once more, our observations align with the findings of Wayda-Zalewska et al. (18), indicating that individuals with AN struggle to maintain psychological balance.

The AN patient exhibited a higher likelihood to engage in oblique transitions, involving changes in arousal and valence. This suggested the requirement of more opposing emotional inputs to facilitate a shift from one emotional state to another. The variability indexes of the patient with anorexia nervosa were diversified: her indexes exhibited substantial variation in some cases and were nearly absent in others, revealing various physiological response patterns based on the emotional transition’s characteristics.

## 5 Conclusion

Emotional difficulties in AN include inappropriate emotion regulation (ER) and expression (66), increased negative affect (NA) in response to unpleasant stimuli (63), and alexithymia (67). Among various emotional experience patterns, NA is critical for the development and maintenance of AN. Studies show that higher daily levels of NA increase the likelihood of food restrictions. NA can also increase following specific AN behaviors such as loss of control (LOC) eating, purging, a combination of LOC and purging, or weight checks. Conversely, NA levels tend to fall after drinking fluids or engaging in physical exercise. Research has focused on the states of NA and their specific manifestations (e.g., anxiety or tension) in AN, along with behaviors such as dietary restriction, LOC, or purging, as well as frequent weighing (18).

To contribute to the advancement of affect dynamics’ assessment research, we introduced a novel experimental and methodological approach that examines cardiac variability, specifically RMSSD, in a clinical and laboratory setting. (68, 69). Using standardized IAPS emotional imagery, researchers can effectively investigate affective transitions in a controlled manner, allowing the detection of cardiac variability parameters.

Using an index like RMSSD, one of the most important indicators for cardiac variability, we could evaluate the short-term variability of the various affective transitions. Furthermore, employing Discrete Time and Discrete Space Markov chains enables us to statistically translate the variability associated with transitions into indexes that show the likelihood of variation between states. The Markov matrix measures descriptive indexes—those calculated on the transition matrix, such as verticals, obliques, horizontals, and trait states—as well as predictive indexes—such as steady states—resulting from the matrix product of the transition matrix and the initial state vector (39). Descriptive indexes provide a snapshot of the patient’s current affective state, while predictive indexes forecast the likelihood of being in one of four affective states after multiple transitions. Empirically, we’re curious about how the participant reacts to an emotional storm caused by the encountered emotional shifts. Will the patient feel stressed, relaxed, or engaged?

The combination of an experimental design with analytical techniques like Markov matrixes creates a patient-centric approach, providing detailed insights into a patient’s affectivity, complementing for a holistic understanding of a patient’s emotional health. Our findings suggest that understanding affect dynamics through a combination of Markov Chains and HRV might improve current therapeutic approaches for treating AN. This would enable the implementation of an emotional assessment that is experimentally simple to measure and clinically simple to interpret for both the clinician and the patient. Indeed, the graph of transitions and steady states are quantitative idiographic methods that provide an instant comprehension of the patient’s emotional dynamism.

The current study has limitations by its nature as a case study, which restricts the generalizability of the observed results. Further studies should employ larger and more varied samples to improve generalizability. Extending this methodology to people with differing severities of AN, comorbidities, or other disorders—such as mood or anxiety disorders—necessitates consideration of condition-specific variations in autonomic and emotional control. Additionally, the absence of assessments on conscious emotional processing, such as explicit questions, hinders our understanding of the specific emotions acknowledged by patients. It would be intriguing to use this component in future research to detect any alignment or disparity between the implicit and explicit regulation aspects. Furthermore, self-report surveys assessing the individual’s emotional regulation styles could be implemented.

Future directions also involve incorporating virtual reality (VR) (70–72) to create dynamic stimuli that can better replicate the transitions and emotional dynamics experienced by individuals in the real world. Future research will also explore the idea of creating tools for the generation and examination of Markov matrixes.

## Data Availability

The dataset used and/or analyzed during the current study is available from the corresponding author on reasonable request.
