# Supplementary material for: A stochastic model for affect dynamics: methodological insights from heart rate variability in an illustrative case of Anorexia Nervosa
Source: Front Psychiatry. 2025 Feb 25;16:1502217. doi: 10.3389/fpsyt.2025.1502217 (PMC11893548; doi:10.3389/fpsyt.2025.1502217)
Supplement: Supplementary file 1 [file DataSheet1.zip › Script/Detailed Documentation for MATLAB Script Structure and Usage.docx]

**Detailed Documentation for MATLAB Script Structure and Usage**

# Overview

This MATLAB script is designed by **Francesca Borghesi** and **Pietro Cipresso** to process and analyze physiological **affect dynamics** data using a **Markov chain model**. The script is flexible, allowing users to adapt it to their experimental design and physiological data.

# Requirements

**Input Data:**

1. **Physiological Data:**
   - Data can include **facial electromyography (EMG)**, **galvanic skin response (GSR)**, or **heart rate variability (HRV)** indices (e.g., RMSSD, SD HR, HR, SDNN, etc.).
   - The data should be stored in a **cell matrix** called Participants with:
     - **Rows:** Number of participants.
     - **Column:** One column containing data for each participant.
2. **Experimental Design:**
   - The script is built for experiments using **IAPS images**, but it can be adapted to other stimuli.
   - Design involves **13 blocks** of images, where each block corresponds to one of the **four basic affective states** from Russell's Circumplex Model:
     - **Stress**
     - **Engagement**
     - **Boredom**
     - **Relaxation**
   - Each block contains **10 images**, with a duration of **12 seconds per image**.
3. **Matrix File:**
   - The provided matrix file contains the **random sequence of the 13 blocks** for each participant.
   - Import the matrix file into MATLAB as a **numeric matrix**.

**Flexibility:**

- Users can modify the **number of transitions** or **state names** by editing the following functions:
  - create_transition_matrix
  - create_self_transition
- Alternatively, users can use the provided script with the pre-configured settings and simply run the **Main_Process** file.

# Steps for Using the Script

1. **Import Your Data:**
   - Insert your physiological data into a cell matrix called Participants.
   - Ensure the data is formatted as:

Participants{1,1} = physiological_data_participant1;

Participants{2,1} = physiological_data_participant2;

...

1. **Import the Matrix File:**
   - Load the matrix.csv file into MATLAB as a numeric matrix:
   - matrix = csvread('matrix.csv');

**Running the Script:**

1. **Main Process Algorithm:**
   - Run the **Main_Process** file.
   - Configure parameters such as (if you want to use our experimental design you DO NOT HAVE TO CHANGE NOTHING (it’s is already in the correct form) in parameters (e.g., epoch division and transition timing), unless the samplig rate):
     - Sampling rate (s)
     - Epoch division
     - Timing for the transition and self-transition windows
2. **Ensure Required Files are in the Same Folder:**
   - Place all supporting .mat files (containing functions) in the same folder as the **Main_Process** file.
3. **Modify Transitions (Optional):**
   - To change the number or names of transitions, edit:
     - create_transition_matrix
     - create_self_transition

**Output:**

The script processes the data and saves the following outputs:

- **Processed Data:**
  - Epoch-divided data stored in the Participants structure.
- **Markov Transition Matrices:**
  - Computed using the **delta matrix**.
  - Stored in a cell array matrix_cell.
- **Steady-State Probabilities:**
  - Calculated for each participant's Markov chain.
  - Stored in steady_ss.

**Notes for Adaptation:**

- If using **different experimental stimuli** or designs, ensure the design is consistent with:
  - 13 blocks for 12 transitions.
  - Appropriate duration and sequence for stimuli.
- For pre-configured settings (e.g., with IAPS images), users only need to:
  - Upload their physiological data.
  - Import the matrix.csv file.
  - Run the **Main_Process** file.

# File Descriptions

**1. Main_Process (main_process.m)**

- Simulates data and defines parameters.
- Calls the main function process_data.

**2. process_data (process_data.m)**

- Core function that calls all other functions sequentially:
  1. epoch: Divides data into baseline, epoch, and post-baseline.
  2. create_transition_matrix: Creates transition matrices based on input data.
  3. create_self_transition: Extracts self-transition indices.
  4. cut_self_transition: Extracts self-transition data.
  5. cut_transition: Extracts transition data based on timing.
  6. extract_transitions: Calculates means for transitions.
  7. extract_self_transition: Calculates means for self-transitions.
  8. create_delta_matrix: Combines transition and self-transition means to form a delta matrix.
  9. create_markov_matrices: Generates 4x4 Markov matrices.
  10. calculate_steady_states: Computes steady-state probabilities for Markov chains.
